# Change of Objectively-Measured Physical Activity during Geriatric Rehabilitation

**DOI:** 10.3390/s19245451

**Published:** 2019-12-11

**Authors:** Jochen Klenk, Sebastian Wekenmann, Lars Schwickert, Ulrich Lindemann, Clemens Becker, Kilian Rapp

**Affiliations:** 1 Department of Clinical Gerontology, Robert-Bosch-Hospital, 70376 Stuttgart, Germany; sebastian.wekenmann@googlemail.com (S.W.); lars.schwickert@rbk.de (L.S.); ulrich.lindemann@rbk.de (U.L.); clemens.becker@rbk.de (C.B.); kilian.rapp@rbk.de (K.R.); 2 Institute of Epidemiology and Medical Biometry, Ulm University, 89081 Ulm, Germany; 3 IB University of Applied Sciences Berlin, Study Center Stuttgart, 70178 Stuttgart, Germany

**Keywords:** accelerometer, wearable sensors, physical activity, geriatric rehabilitation

## Abstract

This prospective study investigated feasibility and sensitivity of sensor-based physical activity (PA) measures to monitor changes in PA during geriatric rehabilitation and its relation to clinical parameters at admission. PA was routinely measured at day 2 and day 15 after admission in 647 patients (70.2% women, mean age = 82.0 (SD = 7.19) years) of a German geriatric hospital using a thigh-worn accelerometer. Clinical records were used to include age, Barthel Index, diagnosis, mobility, orientation and cognition. Mean values and 95% confidence intervals (95%-CI) of walking duration, walking bout duration and number of sit-to-stand transfers were calculated to quantify different domains of PA. All observed PA parameters improved during rehabilitation, regardless of age, diagnosis or physical and cognitive function at admission. Walking duration increased by 12.1 (95%-CI: 10.3; 13.8) min, walking bout duration by 2.39 (95%-CI: 1.77; 3.00) s, and number of sit-to-stand transfers by 7 (95%-CI: 5; 8). Floor and ceiling effects were not observed. Walking duration at day 2 as well as day 15 was continuously associated with Barthel Index and statistically significant improved for all levels of Barthel Index. In summary, this study showed that sensor-based PA monitoring is feasible to assess the individual progress in geriatric rehabilitation patients.

## 1. Introduction

Physical activity is a key component for an active and independent life [1]. It determines three domains: physical capacity, the environment or context and behavioral factors such as motivation and intention. In older persons severe diseases such as a hip fracture or a stroke can lead to functional decline and thereby to a loss of capacity, mobility and independence [2]. Consequently, a major aim of geriatric rehabilitation in patients with these diagnoses is to regain functional capacity, physical activity and independence.

Physical function and capacity are usually assessed during rehabilitation by subjective evaluation of physicians and therapists or standardized clinical tests such as the Timed Up and Go test or the Short Physical Performance Battery [3,4]. These tests and assessments are supervised and focus on capacity rather than unsupervised everyday-life performance. In addition, many patients are not able to perform functional tests at admission due to their disease-specific limitations [5]. This leads to floor effects and missing data. There is also a risk of ceiling effects if tests are not challenging enough, especially at the end of a therapy after a patient has improved [5,6]. Both limitations can affect the interpretation of progress during rehabilitation.

To assess the physical activity domains (capacity and performance) objectively, reliable and sensitive methods are needed. During rehabilitation, physicians have to decide how to use limited resources and time in the best interest of the patient and the health system [7]. A precise measurement of physical activity could be used for goal setting, to give clinicians guidance on the physical status of a patient, to readjust therapy and to estimate the remaining demands to reach a sufficient level of activity [8]. It also enables an objective documentation of the rehabilitation progress, indicates whether the patient is able to live independently after discharge and provides evidence for the need of an extension of the rehabilitation. This may be useful for a more objective communication between the rehabilitation clinic and the patient’s health insurance in the future [9,10].

Previous research has shown that self-reports of physical activity are problematic. Particularly in populations with a high prevalence of cognitive impairment, recalling activities can lead to information bias [11]. With the development of small, robust and cost-effective inertial sensors during the last decade, physical activity can be objectively measured using body-fixed devices [12]. Besides the cumulative amount of physical activity, sensor data enables to extract further information about different aspects of activity, such as walking bout duration or sit-to-stand transfers [13], which can support diagnosis and decision making in geriatric rehabilitation [14].

Although inertial sensor devices have been widely used in cohort studies with community-dwelling older persons, there are only few studies in patients during geriatric rehabilitation [8,15,16,17,18,19]. These studies had small sample sizes, showed inconsistent results and were not implemented in the clinical routine to document the progress during rehabilitation.

In this context it is important to understand how the progress in physical activity is associated with other routine clinical parameters which are used to assess and tailor geriatric rehabilitation such as age or Barthel Index at admission as a measure of performance of daily activity [20,21].

Therefore, the aim of this study was to investigate the feasibility and sensitivity of objective sensor-based physical activity measures to document changes in physical activity during geriatric rehabilitation and its relation to clinical parameters at admission such as age and Barthel Index.

## 2. Materials and Methods

For this prospective cohort study, physical activity at day 2 and day 15 after admission was routinely measured between July 2012 and February 2014 in 1,251 patients of a geriatric rehabilitation clinic in Southern Germany. Clinical records were used to include age, Barthel Index, diagnosis, mobility, orientation and cognition at admission. All patients received usual care consisting of physiotherapy, occupational therapy and training therapy. Therapy was offered as individual sessions and group training (15–20 therapy sessions per week).

For the present analysis patients with missing data on day 2 (no or later measurement) were excluded (n = 220). In addition, physical activity data on day 15 was not available for 335 patients. Finally, patients with incomplete 24 h measurements and missing clinical characteristics were excluded (n = 49). The final study sample consisted of 647 patients.

As data was recorded during clinical routine and analyzed fully anonymized, study-specific written informed consent was not necessary (the treatment contract covered the use of data for anonymized analyses). The Ethics Committee of the University of Tübingen approved the study (application no. 241/2016BO1).

### 2.1. Clinical Characteristics

To analyze the effect of patient characteristics at admission on the change of physical activity during rehabilitation several variables from clinical records were obtained. Age was analyzed continuously and categorized in four groups (<70 years, 70–79 years, 80–89 years, ≥90 years). Diagnosis was categorized in three groups: femur fractures (ICD-10: S72), stroke (ICD-10: I64) and others. The Barthel Index as a measure of activities of daily living was assessed by nursing staff [20]. It consists of 10 domains including nutrition, personal hygiene and mobility. Values range from 0 (fully dependent) to 100 (fully independent). Barthel Index was analyzed continuously as well as categorized into two groups (<50 vs. ≥50). Orientation disorder of each patient, including time and place, was rated at admission by nursing staff (fully orientated vs. orientation disorder). Furthermore, nursing staff rated the mobility of each patient at admission according to the degree of support needed (1: walking not possible/wheelchair dependent, 2: walking possible with staff support, 3: walking possible independently including the use of walking aids). Cognition was assessed by the DemTect, a screening test assessing memory, verbal fluency, executive function and attention (scores range from 0 (worst) to 18 (best)) [22]. Patients were grouped in three categories (0–8: dementia, 9–12: mild cognitive impairment, 13–18: adequate cognitive performance).

### 2.2. Physical Activity

Physical activity was measured using a validated three-axial accelerometer (activPAL3, PAL Technologies Ltd., Glasgow, UK) [23,24,25]. Compared to other devices, the activPAL3 has a high accuracy (between 96% and 100% [24,25]) to detect different postures and transfers due to the sensor location at the thigh. The absolute percentage error of walking duration ranged from below 5% up to 40% in persons with a very low gait speed (<0.47 m/s) [23,25]. The device was attached to the thigh using waterproof adhesive tape. Both, the selected sensor location and the attachment method showed a good compliance in previous studies, especially in the target population. Participants were instructed to wear the sensor over 48 h, which included one complete day of measurement (24 h). For the first assessment the sensor was attached at the first day during rehabilitation, measuring physical activity at admission to the geriatric rehabilitation hospital (at day 2). The follow-up assessment was conducted 14 days after the first measurement at day 15 to assess the change of physical activity during rehabilitation. This is an important time point during rehabilitation to evaluate the individual progress and to adjust therapy or to apply for an extension of the rehabilitation if needed. If it was not possible to measure physical activity at day 15, e.g., due to a weekend, day 16 or day 14 were used for analysis. If an assessment day was a public holiday the next working day was measured.

The data processing algorithm detects upright posture as well as walking patterns and classifies the activity into three categories: (1) lying or sitting, (2) standing and (3) walking (including low to high intensity walking). To describe the physical activity patterns, the following parameters were calculated: daily cumulative walking duration (in minutes), average walking bout duration (in seconds), and number of sit-to-stand transfers. A walking bout was defined as the interval between two periods of standing.

### 2.3. Statistics

For each category of clinical variables mean values with 95% confidence intervals (95%-CI) for each physical activity variable was calculated for day 2 and day 15 as well as for the change between day 2 and day 15.

Furthermore, we continuously evaluated the nonlinear association of age and Barthel Index with walking duration at day 2 and day 15 as well as its change using restricted cubic splines with knots at the 5, 35, 65, and 95%, respectively. To reduce the effect of extreme values on the margins of the distribution we excluded 2.5% of the observations on each margin for the presentation. Splines were adjusted for sex and either for Barthel Index or age. All analyses were performed using SAS 9.4.3.

## 3. Results

The study population consisted of 647 subjects (70.2% women) with a mean age of 82.0 (SD = 7.19) years. Femoral fractures showed the highest prevalence with 22.4% and cognitive impairment was present in about 60% of all patients (Table 1). The average duration of the rehabilitation was 23.4 (SD = 6.3) days.

Table 2 shows the association between clinical parameters at admission, physical activity variables at day 2 and day 15 as well as the change of physical activity variables. All parameters improved statistically significant during rehabilitation between day 2 and day 15. Walking duration increased by 12.1 (95%-CI: 10.3; 13.8) min, walking bout duration by 2.39 (95%-CI: 1.77; 3.00) s, and number of sit-to-stand transfers by 7 (95%-CI: 5; 8).

While age did not show a consistent relation with walking duration and walking bout duration at admission a clear negative dose-response association was observed at day 15 for both variables. In contrast, there were no associations between age and number of sit-to-stand transfers.

Patients with a Barthel Index below 50 at admission improved less in walking duration (9.4 (95%-CI: 7.0; 11.7) min.) compared to those with higher values (12.9 (95%-CI: 10.8; 15.0) min.). Conversely, a low Barthel Index at admission was associated with a higher positive change in walking bout duration and number of sit-to-stand transfers.

For patients with a femur fracture the improvement in the number of sit-to-stand-transfers was twice as high compared to cerebrovascular diseases or other diagnoses.

The Mobility Index was closely related to all physical activity parameters at all stages of rehabilitation. However, there remained a larger gap between Mobility Index group 1 and the other Mobility Index groups for walking duration and walking bout duration at day 15. Looking at the change of walking parameters, Mobility group 1 did improve less in walking duration compared to Mobility group 2 and 3, while the improvement in walking bout duration was negatively associated with Mobility Index in a dose-response relationship.

Orientation and cognition (DemTect) at admission did not show any effects with physical activity parameters.

**Figure 1:**
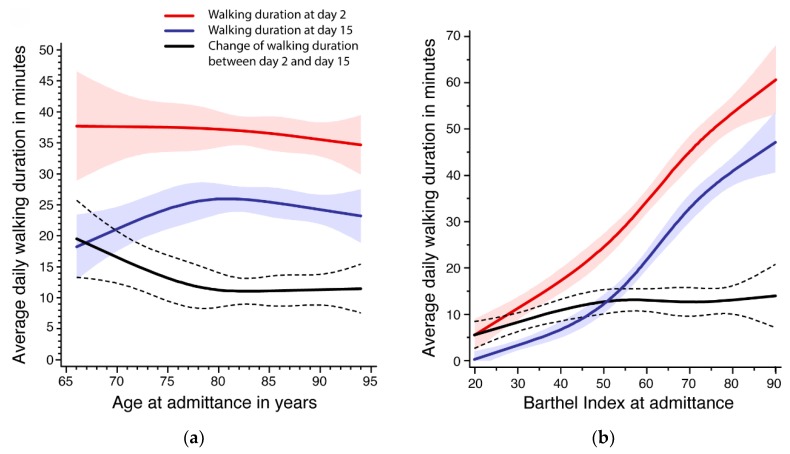
Association between age (**a**) and Barthel Index (**b**) at admission (day 2) with average walking duration at day 2 and day 15 as well as the change of average daily walking duration between day 2 and day 15.

The evaluation of the nonlinear association of age and Barthel Index with walking duration is presented in Figure 1. Below the age of 80 years, age was negatively associated with walking duration improvement (Figure 1a). This was mainly due to lower activity levels at admission. In contrast, older patients had a similar improvement of about 10 min between day 2 and day 15. The average activity level at day 15 was similar for all age groups.

Figure 1b shows a clear positive association between Barthel Index at admission and walking duration at day 2 as well as day 15, and a statistically significant improvement in walking duration for all levels of Barthel Index. Between a Barthel Index of 20 and 50, the change of walking duration increased from about 5 to 12 min and remained nearly constant after 50.

## 4. Discussion

The results of our study showed that all patients benefited from geriatric rehabilitation according to walking duration, walking bout duration and number of sit-to-stand transfers, regardless of age, diagnosis group or physical and cognitive function at admission. Floor and ceiling effects were not observed.

Our findings are in line with previous studies [8,15,16,18,19] although the observed effects varied and outcomes were limited to walking duration and up-time. Two studies in different disease groups were performed from 2008–2010 in the same geriatric hospital as the present study with a similar design but a much smaller sample size and a different sensor system [15,16]. In both previous studies the amount of walking time was systematically lower, which might be due to the different sensor setup with a sensor location on the chest and a limited wear-time of 9 h. In femoral fracture patients the relative increase of walking duration during rehabilitation was comparable at about 100% [16], while in stroke patients the relative increase was lower in the previous study (21.9% vs. 37.5%) [15].

Three studies used the same sensor system as in the current study, two of them evaluated daily walking duration [8,18] and one study used upright duration as the outcome measure [19]. The results were inconsistent, reflecting much smaller sample sizes, different study designs and settings. The control group of an intervention study in a geriatric rehabilitation hospital in Australia showed a similar improvement in walking duration of about 6 min per week of rehabilitation [8]. However, walking duration during therapy was excluded. A study in an acute geriatric ward showed very low physical activity values at baseline with a median walking duration of 4 min. per day [18]. Time spent walking increased until the fourth quartile of rehabilitation up to a median of 10 min. per day. The study population seemed to be very frail and might be similar to those in the current study in Mobility Index group 1. Kronborg et al. reported an increase of upright duration as a measure of physical activity during the first 7 days after hip fracture surgery [19]. Although the reported effect is similar to the other studies, a direct comparison is not possible due to the different outcome variable.

The primary aim of rehabilitation is to increase participation of patients, which is closely related to activity according to the International Classification of Functioning, Disability and Health (ICF). Currently, this aspect is not routinely assessed in clinical practice. Measuring physical activity bridges the gap and provides important information for clinicians. It can be used to objectively define and examine rehabilitation targets together with the patient, to individually adjust therapy and to improve the communication with the patient. Physicians and therapist can also assess the activity levels outside the therapy sessions, which might better reflect everyday life abilities and intrinsic motivation.

The results of the present study provide the average association between age, Barthel Index and physical activity during rehabilitation. This information can help clinicians to compare the individual progress of a patient with the expected outcome depending on the current functional status. During weekly team meetings, for example, such objectively measured data might support decisions, which are currently often based on more subjective observations of therapists and physicians. Furthermore, this information can be used to provide the health insurance company an objective justification for a rehabilitation extension if needed.

In contrast to previous studies, the current analyses focused on several aspects of physical activity. Splitting physical activity in different domains such as walking duration, walking bout duration and number of sit-to-stand transfers enables a deeper insight in the complex construct of physical activity.

In the current study it seems that patients with low mobility at admission (Mobility Index 1) benefit only marginally on the walking duration domain. However, they improved on the walking bout duration and sit-to-stand domains. The same pattern was observed for patients with a low Barthel Index at admission. This might reflect that regaining physical activity is obtained via different domains of activity, starting with sit-to-stand transfers, followed by an increase of walking bout duration and finally by an increase in walking duration. Sensor-based measurement of patients in geriatric rehabilitation might enable the analysis of such patterns in future studies and the monitoring in the clinical routine.

There is also a great potential to extract further meaningful functional parameters from sensor data. Recent studies demonstrated that the sit-to-stand transfer can be used to measure lower limb muscle strength and power [26,27]. Sensor-based data related to physical activity and function will further improve diagnosis, therapy and rehabilitation and become increasingly important in healthcare as digital biomarkers. This is also reflected by a large ongoing EU-funded ‘Innovative Medicines Initiative’ project focusing on digital mobility outcomes for clinical studies (MOBILISE-D) [28]. Furthermore, future studies will have to analyze the change of physical activity after discharge from rehabilitation to the home setting. It is important to understand the prognostic value of in-clinic physical activity measures for an independent everyday life.

### Strengths and Limitations

The major strengths of our study are the large number of patients and the objective measurement of physical activity at day 2 and day 15 of the rehabilitation period. Especially in older patients, including cognitively impaired persons, sensor-based measurements can improve the assessment of physical activity considerably. If the activity levels are low, daily activity is mostly accumulated by short and light activity intervals, which is more difficult to memorize and count. Furthermore, patients showed a very high compliance with the activPAL device and its sensor location at the thigh. Compared to other devices worn on the lower back, this location is unobtrusive and does not lead to ulcers in hospital patients lying a considerable percentage of the day in bed. Moreover, the sensor location of the activPAL enables a very precise assessment of transfers, which is an important but rarely investigated physical activity outcome, especially in geriatric rehabilitation patients.

The main limitation of the study was the exclusion of patients due to missing data. To increase comparability the inclusion criteria regarding the assessment period were very strict. However, it seems that the exclusion was at random and should not have had a strong effect on the results. In this context, the fact that physical activity was only measured twice for 24 h during rehabilitation may be also considered a limitation of the study. Due to a limited availability of devices and to improve compliance we decided to measure physical activity twice for 24 h. A continuous assessment might have reduced the exclusion of patients and improved the precision of the results. There is evidence that the increase of physical function is not equally distributed across the rehabilitation period, which might also be the case for the change of physical activity [29]. With the ongoing technical development, the increase of measurement duration, the reduction of sensor size and costs a continuous monitoring of physical activity during the whole hospitalization will be very likely in the future. Furthermore, daily routine in a geriatric rehabilitation clinic is very structured and physical activity is partly predetermined by the individual therapy plan. It was not possible to differentiate between activities related to therapy and individually initiated activities. It would be interesting to investigate the effect of external factors on physical activity in future studies and to analyze activities also on weekends without structured therapies. Although accelerometry seems to be one of the most reasonable methods to quantify physical activity [11], a decrease in detection-sensitivity was observed at slow walking speed [23,25]. This might have underestimated walking duration and walking bout duration especially at admission when gait speed is likely to be lower compared to day 15. Finally, the system of geriatric rehabilitation is very specific for Germany. The one-to-one transferability of the results to other health systems might be limited and has to be proven for other settings.

## 5. Conclusions

In conclusion, this study showed that sensor-based physical activity monitoring in German geriatric rehabilitation patients is a feasible method to assess individual progress without floor and ceiling effects. All patients benefited from geriatric rehabilitation and improved their physical activity at a statistically significant level. Different measurements from sensor data reflect different domains of physical activity and provide detailed information for clinicians about the progress and the individual needs of their patients.

## Figures and Tables

**Table 1:** Characteristics of the study population.

	Total n = 647	Womenn (%) = 454 (70.2)	Menn (%) = 193 (29.8)
Age [years], mean (SD)	82.0 (7.19)	82.9 (6.98)	79.7 (7.22)
<70 years, n (%)	33 (5.1)	16 (3.5)	17 (8.8)
70–79 years, n (%)	198 (30.6)	121 (26.7)	77 (39.9)
80–89 years, n (%)	322 (49.8)	236 (52.0)	86 (44.6)
>89 years, n (%)	94 (14.5)	81 (17.8)	13 (6.7)
Barthel Index at admission, mean (SD)	61.1 (18.1)	62.6 (16.8)	57.6 (20.3)
0–<50 Barthel Index, n (%)	132 (20.4)	72 (15.9)	60 (31.1)
≥50 Barthel Index, n (%)	514 (79.6)	381 (84.1)	133 (68.9)
Diagnosis at admission			
Femur fracture, n (%)	145 (22.4)	112 (24.7)	33 (17.1)
Cerebrovascular disease, n (%)	58 (9.0)	37 (8.2)	21 (10.9)
Other, n (%)	444 (68.6)	305 (67.2)	139 (72.0)
Mobility at admission			
walking not possible, n (%)	80 (12.6)	50 (11.3)	30 (15.6)
walking possible with staff support, n (%)	179 (28.2)	119 (26.9)	60 (31.3)
walking possible independently, n (%)	376 (59.2)	274 (61.9)	102 (53.1)
Orientation at admission			
Orientation disorder, n (%)	151 (23.7)	99 (22.3)	52 (27.2)
No orientation disorder, n (%)	485 (76.3)	346 (77.8)	139 (72.8)
DemTect at admission, mean (SD)	10.8 (4.1)	11.0 (4.1)	10.1 (4.0)
0–8 DemTect, n (%)	174 (30.8)	120 (29.9)	54 (33.1)
9–12 DemTect, n (%)	181 (32.0)	119 (29.6)	62 (38.0)
13–18 DemTect, n (%)	210 (37.2)	163 (40.6)	47 (28.8)
Duration of rehabilitation [days], mean (SD)	23.4 (6.3)	23.3 (5.9)	23.8 (7.2)
Duration between 1. and 2. assessment			
13 days, n (%)	15 (2.3)	12 (2.6)	3 (1.6)
14 days, n (%)	575 (88.9)	401 (88.3)	174 (90.2)
15 days, n (%)	57 (8.8)	41 (9.0)	16 (8.3)

**Table 2:** Association between clinical variables at admission and change of physical activity measures during geriatric rehabilitation between day 2 (t_1_) and day 15 (t_2_).

	N	Mean daily Walking Duration in Minutes (95%-CI)				Mean Walking Bout Duration in Seconds (95%-CI)				Mean Number of STS Transfers (95%-CI)		
		t_1_	Change t_1-2_	t_2_		t_1_	Change t_1-2_	t_2_		t_1_	Change t_1-2_	t_2_
Total	647	24.6 (22.7; 26.5)	12.1 (10.3; 13.8)	36.6 (34.3; 38.9)		12.5 (11.7; 13.4)	2.39 (1.77; 3.00)	14.6 (14.0; 15.1)		48 (46; 50)	7 (5; 8)	55 (53; 56)
Age												
<70 years	33	19.3 (10.4; 28.3)	21.5 (13.1; 30.0)	40.9 (27.3; 54.4)		12.5 (9.4; 15.5)	2.70 (−0.44; 5.83)	15.5 (12.8; 18.1)		43 (33; 52)	9 (0.03; 17)	51 (43; 60)
70–79 years	198	25.8 (21.9; 29.6)	12.6 (8.8; 16.5)	38.4 (33.6; 43.3)		12.3 (11.4; 13.2)	2.50 (1.47; 3.53)	14.9 (13.9; 15.9)		48 (45; 51)	7 (4; 9)	55 (51; 58)
80–89 years	322	26.1 (23.5; 28.6)	10.9 (8.7; 13.2)	37.0 (34.1; 39.9)		13.1 (11.6; 14.6)	2.40 (1.45; 3.34)	14.8 (14.1; 15.6)		50 (47; 52)	6 (4; 8)	56 (53; 58)
>89 years	94	18.8 (14.4; 23.2)	11.3 (7.6; 15.0)	30.1 (25.0; 35.2)		11.1 (10.0; 12.3)	1.99 (0.69; 3.29)	12.8 (11.5; 14.1)		43 (39; 47)	9 (5; 12)	52 (47; 56)
Barthel Index at admission												
0–<50 Barthel Index	132	3.9 (2.7; 5.1)	9.4 (7.0; 11.7)	13.3 (10.3; 16.2)		10.9 (7.2; 14.6)	3.81 (1.82; 5.81)	12.8 (11.1; 14.4)		25 (22; 28)	9 (6; 12)	34 (31; 38)
≥50 Barthel Index	514	29.8 (27.6; 31.9)	12.9 (10.8; 15.0)	42.7 (40.2; 45.2)		12.9 (12.4; 13.4)	2.06 (1.47; 2.66)	15.0 (14.5; 15.6)		53 (52; 55)	6 (5; 8)	60 (58; 62)
Diagnosis at admission												
Femur fracture	145	15.2 (12.2; 18.3)	13.0 (9.2; 16.9)	28.3 (23.9; 32.7)		10.4 (9.4; 11.5)	2.81 (1.51; 4.11)	13.1 (12.0; 14.2)		39 (35; 42)	11 (8; 13)	50 (46; 53)
Cerebrovascular disease	58	34.1 (27.2; 41.0)	12.8 (7.3; 18.2)	46.8 (39.0; 54.61)		14.0 (12.1; 15.9)	1.88 (−0.37; 4.14)	16.2 (14.4; 18.1)		53 (46; 59)	5 (2; 8)	57 (51; 64)
Other	444	26.4 (24.0; 28.7)	11.7 (9.5; 13.8)	38.0 (35.2; 40.9)		13.0 (11.9; 14.1)	2.32 (1.58; 3.05)	14.8 (14.2; 15.5)		50 (48; 52)	6 (4; 7)	56 (53; 58)
Mobility at admission												
walking not possible, n (%)	80	1.3 (0.7; 1.9)	6.3 (3.8; 8.9)	7.7 (4.9; 10.4)		7.3 (5.3; 9.3)	4.75 (1.66; 7.85)	11.5 (9.2; 13.9)		22 (18; 25)	8 (5; 11)	30 (26; 34)
walking possible with staff support, n (%)	179	12.0 (9.7; 14.3)	13.3 (10.2; 16.5)	25.4 (21.7; 29.0)		11.2 (10.2; 12.2)	2.75 (1.44; 4.06)	14.1 (13.0; 15.2)		38 (36; 41)	10 (7; 13)	49 (45; 52)
walking possible independently, n (%)	376	35.2 (32.7; 37.7)	13.0 (10.5; 15.5)	48.2 (45.3; 51.2)		14.3 (13.0; 15.5)	1.77 (1.16; 2.37)	15.5 (14.9; 16.1)		57 (55; 60)	5 (3; 7)	62 (60; 65)
Orientation at admission												
Orientation disorder	151	20.9 (17.2; 24.6)	12.0 (8.8; 15.2)	32.9 (28.2 (37.5)		12.0 (10.9; 13.1)	2.42 (1.15; 3.69)	14.1 (12.8; 15.3)		44 (40; 48)	9 (6; 11)	52 (48; 57)
No orientation disorder	485	25.7 (23.5; 28.0)	12.3 (10.2; 14.4)	38.0 (35.3; 40.7)		12.7 (11.7; 13.8)	2.42 (1.71; 3.13)	14.8 (14.2; 15.4)		49 (47; 51)	6 (5; 8)	55 (53; 57)
DemTect at admission												
0–8 DemTect	174	23.6 (19.9; 27.3)	13.2 (9.6; 16.8)	36.8 (31.8; 41.8)		13.2 (10.6; 15.8)	3.21 (1.88; 4.55)	15.2 (14.0; 16.4)		45 (42; 49)	7 (4; 10)	52 (49 (56)
9–12 DemTect	181	28.3 (24.5; 32.1)	9.7 (6.3; 13.2)	38.1 (34.0; 42.1)		12.9 (12.0; 13.7)	1.63 (0.63; 2.64)	14.4 (13.5; 15.4)		52 (48; 55)	6 (3; 9)	58 (55; 62)
13–18 DemTect	210	23.3 (20.1; 26.5)	14.0 (11.3; 16.7)	37.3 (33.6; 41.0)		12.1 (11.1; 13.1)	2.32 (1.26; 3.39)	14.4 (13.6; 15.2)		48 (45; 51)	8 (6; 10)	56 (53; 58)

95%-CI: 95%-confidence interval, DemTect: cognition screening test, STS: sit-to-stand.
